# Genetic variation in the insulin, insulin-like growth factor, growth hormone, and leptin pathways in relation to breast cancer in African-American women: the AMBER consortium

**DOI:** 10.1038/npjbcancer.2016.34

**Published:** 2016-10-26

**Authors:** Edward A Ruiz-Narváez, Kathryn L Lunetta, Chi-Chen Hong, Stephen Haddad, Song Yao, Ting-Yuan David Cheng, Jeannette T Bensen, Elisa V Bandera, Christopher A Haiman, Melissa A Troester, Christine B Ambrosone, Lynn Rosenberg, Julie R Palmer

**Affiliations:** 1 Slone Epidemiology Center at Boston University, Boston, MA, USA; 2 Department of Biostatistics, Boston University School of Public Health, Boston, MA, USA; 3 Department of Cancer Prevention and Control, Roswell Park Cancer Institute, Buffalo, NY, USA; 4 Department of Epidemiology, Gillings School of Global Health, University of North Carolina at Chapel Hill, Chapel Hill, NC, USA; 5 Rutgers Cancer Institute of New Jersey, New Brunswick, NJ, USA; 6 Department of Preventive Medicine, Keck School of Medicine, University of Southern California/Norris Comprehensive Cancer Center, Los Angeles, CA, USA

## Abstract

The insulin/insulin-like growth factor (IGF) system and related pathways such as growth hormone, and leptin signaling have a key role in cancer development. It is unclear how germline variation in these pathways affects breast cancer risk. We conducted gene-based analyses of 184 genes in the insulin/IGF, growth hormone, and leptin pathways to identify genetic variation associated with risk of breast cancer overall, and for estrogen receptor (ER) subtypes. Tag single-nucleotide polymorphisms (SNPs) for each gene were selected and genotyped on a customized Illumina SNP array. Imputation was carried out using 1000 Genomes haplotypes. The analysis included 91,627 SNPs genotyped or imputed in 3,663 breast cancer cases, (1,983 ER-positive and 1,098 ER-negative) and 4,687 controls from the African American Breast Cancer Epidemiology and Risk consortium, a collaborative project of four large studies of breast cancer in African-American women (Carolina Breast Cancer Study, Black Women's Health Study, Women's Circle of Health Study, and Multiethnic Cohort). We used a multi-locus adaptive joint test to determine the association of each gene with overall breast cancer and ER subtypes. The most significant gene associations (*P*⩽0.01) were *BAIAP2* and *CALM2* for overall breast cancer; *BAIAP2* and *CSNK2A1* for ER^+^ breast cancer; and *BRAF*, *BAD*, and *MAPK3* for ER^−^ breast cancer. The association of *BAD* with ER^−^ breast cancer was explained by a two-SNP risk model; all other associations were best explained by one-SNP risk models. In total, six genes and seven SNPs had suggestive associations with overall breast cancer or ER subtypes in African-American women.

## Introduction

A growing body of evidence shows that the insulin signaling system has a key role in cancer development and progression. Excess body weight, a condition leading to insulin resistance and hyperinsulinemia, is a recognized risk factor for postmenopausal breast cancer.^1–3^ Although the association between body weight and breast cancer is mediated in part by higher levels of estrogen in overweight women, insulin levels seems to have a larger mediating role.^4^ Recent results show that high levels of insulin levels rather than adiposity is the relevant risk factor in relation to breast cancer risk. Overweight women with low insulin levels have no elevated risk of breast cancer compared with normal-weight women with low insulin levels, and women with high insulin levels have elevated risk of breast cancer irrespective of their body weight.^5^

Insulin-like growth factor 1 (IGF-1) is a hormone with extensive sequence homology to insulin. In addition, IGF-1 and insulin share downstream signaling pathways. Circulating levels of IGF-1 have been found to be positively associated with breast cancer risk.^6–9^ A pooled analysis of 17 prospective studies found that the association of circulating IGF-1 with breast cancer was not modified by circulating levels of IGF-binding protein 3 (IGFBP-3) (i.e., the major protein carrier of IGF-1 in circulation), and seems to be specific to estrogen-positive tumors.^10^

Other signaling pathways (e.g., growth hormone and leptin) interact with the insulin/IGF-1 system to modulate insulin/IGF signaling. Growth hormone (GH) released from the pituitary gland stimulates production and release of IGF-1 from the liver, and elevated levels of circulating GH leads to insulin resistance and hyperinsulinemia.^11,12^ Although there is no evidence linking circulating GH levels with breast cancer risk,^13,14^ other lines of research suggest a role of the GH signaling pathway in breast cancer. For example, expression of the *GH* gene in breast epithelial cells is associated with the presence of proliferative disorders of the mammary gland;^15^ the GH receptor (*GHR*) gene has higher expression in breast tumors compared to adjacent normal breast tissue;^16^ and deficiency of GHR due to splice and nonsense mutations in the *GHR* gene results in a drastic reduction of risk of any type of cancer.^17^ Leptin (LEP) signaling also interacts with the insulin/IGF-1 system and may affect risk of breast cancer. Higher circulating levels of LEP have been found associated with breast cancer risk.^18,19^ It is noteworthy that although the *LEP* and LEP receptor (*LEPR*) genes have a very-low expression in normal breast tissue, both genes are highly expressed in breast tumor ^20,21^ in response to high levels of estrogens, insulin, and IGF-1.^21^

Although circulating levels of insulin, IGF-1, GH and LEP in relation to breast cancer have been well studied, less is known about how germline variation in the insulin, IGF, GH, and LEP signaling pathways may affect risk of breast cancer. Several studies have shown that circulating levels of IGF-1 and IGFBP-3 are predicted by genetic variation in the *IGF-1* and *IGFBP-3* genes.^22–24^ However, genetic variation in the insulin, IGF, GH, and LEP pathways has not been found to be associated with risk of breast cancer.^24,25^ For example, the Breast and Prostate Cancer Cohort Consortium (BPC3) did not find evidence of association of genetic variants in the insulin and IGF pathways with breast cancer after assessing common genetic variation in 24 genes in >6,000 cases of breast cancer and >8,000 controls.^25^ Associations of genetic variants in the *LEP, LEPR*, *GHs*, and *GHR* genes with breast cancer are inconsistent.^26–29^ Moreover, most of these previous studies have been conducted in women of European ancestry.

To assess whether genetic variation in the insulin, IGF, GH, and LEP pathways affect risk of breast cancer in African-American women, we conducted gene-based analysis of 184 genes in these pathways in the African American Breast Cancer Epidemiology and Risk (AMBER) consortium in relation to overall risk of breast cancer, and ER^+^ and ER^−^ breast cancer subtypes.

## Results

Table 1 shows the distribution of subtypes and age at diagnosis among cases by study site. A total of 3,663 breast cancer cases (1,983 ER^+^ cases, 1,098 ER^−^ cases, and 582 unknown ER status) and 4,687 controls were included in the present analysis.

None of the tested genes was significantly associated with overall, ER^+^, or ER^−^ breast cancer after adjustment for multiple testing (α=3.0×10^−4^, Supplementary Table 1). Table 2 shows genes associated with at least one of the outcomes at a less stringent significance level of α=0.01. Two genes, *BAIAP2* and *CALM2*, were associated with overall breast cancer. *BAIAP2* and *CSNK2A1* were associated with ER^+^ breast cancer, and *BRAF*, *BAD* and *MAPK3* were associated with ER^−^ breast cancer.

Table 3 shows the SNPs that best explain the observed gene–disease associations. With the exception of *BAD*, a one-SNP model provided the best fit for the association of each gene with breast cancer. Rs142882938, a deletion/insertion variation (−/T), explained the association of *BAIAP2* with all breast cancer and ER^+^ breast cancer. The frequency of the deletion was 4.8% in AMBER controls and ORs were 1.45 (*P*=6.0×10^−7^) for overall breast cancer, 1.55 (*P*=4.6×10^−7^) for ER^+^ breast cancer, and 1.39 (*P*=3.4×10^−3^) for ER^−^ breast cancer. For *CALM2*, ORs for SNP rs13032512, with a risk-allele frequency of 5.5% in AMBER controls, were 1.33 (*P*=1.3×10^−4^), 1.30 (*P*=4.1×10^−3^), and 1.35 (*P*=8.2×10^−3^) for overall, ER^+^, and ER^−^ breast cancer, respectively. The association between *CSNK2A1* with ER^+^ breast cancer was explained by SNP rs434410. The C-allele has 24.3% frequency in AMBER controls, and was associated with higher risk of overall, ER^+^, and ER^−^ breast cancer.

The other three genes were associated with ER^−^ breast cancer only. A SNP in *BRAF* (rs114729114) showed an OR of 2.04 (*P*=4.9×10^−6^) for ER^−^ breast cancer. Weaker associations were also observed for overall, and ER^+^ breast cancer. For *BAD*, a two-SNP model (rs2286615 and ch11:64038448:I, *r*^2^=0.002 between the two variants) was the best fit. Rs2286615 (minor-allele frequency (MAF) of 4.3%) had an OR of 0.60 (*P*=5.0×10^−4^) for ER^−^ breast cancer, and the minor allele of ch11:64038448:I, a deletion, showed an OR of 0.70 (4.4×10^−3^) for ER^−^ breast cancer. Finally, a SNP in *MAPK3* (rs78564187) was associated with ER^−^ breast cancer, OR equal to 1.26 (*P*=3.7×10^−4^) per high-risk allele.

## Discussion

In this large gene-based analysis of the insulin, IGF, GH, and LEP pathways no genes were associated with breast cancer risk after adjustment for multiple testing, but six genes carried genetic variations showing moderate to strong associations (OR>1.2 or <0.6) for breast cancer overall or an ER-defined subtype, with *P* values<0.01. *BAIAP2* was associated with overall and ER^+^ breast cancer; *CALM2* with overall breast cancer; *CSNK2A1* with ER^+^ breast cancer; and *BRAF, BAD,* and *MAPK3* with ER^−^ breast cancer.

The insulin, IGF, GH and LEP pathways are well-characterized in the biological literature, and previous literature suggests important functions or potential functions for each of these genes in breast cancer. Namely, *BAIAP2* codes the adaptor protein IRSp53, which functions as a substrate of the insulin receptor and IGF-1 receptor tyrosine kinases,^30^ and links membrane bound small GTPases such as Rac1 to trigger re-organization of the cytoskeleton (reviewed in ref. 31). *In vitro* studies have shown that activation of Rac1 promotes metastatic behavior of breast cancer cells.^32,33^ *CALM2* is a member of the gene family (*CALM1*, *CALM2*, and *CALM3*) that encodes the calcium-binding protein calmodulin, involved in cell growth, differentiation, proliferation, and survival.^34,35^ *CSNK2A1* codes a serine/threonine kinase (CK2) that participates in diverse signaling pathways involved in control of the cell cycle, and apoptosis among other cellular processes.^36^ *BRAF* codes a protein member of the family of Raf serine/threonine kinases that regulate signaling of the MAPK pathway. *BAD* codes a protein member of the BCL-2 family that regulates programmed cell death and whose proapoptotic activity is regulated by the PI3K/Akt pathway. High levels of phosphorylated BAD (pBAD) have been found associated with development and progression of ovarian, breast, colon, and endometrial cancer.^37^ *MAPK3* codes a protein that is member of the mitogen-activated protein (MAP) kinase family that participates in the Ras/Raf/MAPK pathway. Expression of *MAPK3* is dysregulated in several cancers including breast.^38,39^

No *CSNK2A1* SNPs have been previously reported associated with breast cancer. A GWAS in German subjects identified rs6038071 (*r*^2^=0.001 with rs434410 in African ancestry populations from 1000 Genomes), 40 kb upstream of *CSNK2A1*, to be associated with familial colorectal cancer.^40^ *BRAF* is usually amplified in somatic DNA from basal-like breast cancers,^41^ but to our knowledge, germline variation in *BRAF* has not been associated with breast cancer. There is also a lack of evidence from previous literature for an association of breast cancer risk with the other SNPs examined.

The present work is a comprehensive assessment of the insulin, IGF, GH, and LEP pathways. Previous studies have partially addressed these pathways, but none have included all of the relevant genes. The Breast and Prostate Cancer Cohort Consortium (BPC3) assessed common variation in 24 genes in the insulin and IGF pathways in European ancestry women and found no single-SNP associated with breast cancer using a threshold of *P*<4.7×10^−5^ to adjust for the total number of tested SNPs.^25^ Although in BPC3 gene variants in *IGF1* and *SSTR5* were associated with circulating levels of IGF-1, and SNPs in *IGFBP3* and *IGFALS* were associated with circulating levels of IGFBP-3, these variants only explained a small fraction of the variation of IGF-1 and IGFBP-3 circulating levels.^42^

The present study has several strengths, including its large size, information on ER subtypes, and large number of genes and SNPs evaluated in the insulin, IGF, GH, and LEP pathways. Although >90 independent loci have been identified that explain about 16% of the familial risk of breast cancer, most of these variants have been established in Europeans and East Asian populations.^43,44^ Thus, present findings add to our understanding of the etiology of breast cancer in African-American women**.** However, we do note some limitations. Most of the SNPs of interest were imputed, although we restricted our analyses to SNPs with high imputation scores and MAFs of at least 2% to minimize imputation errors. Also, we did not examine gene–gene interactions due to limited power even with our large study population.

In summary, our findings suggest that variation in genes in the insulin, IGF, GH, and LEP pathways contribute to the risk of breast cancer and, in particular, to ER-negative breast cancer in African-American women. Because the strength of these associations was moderate for individual genes, future studies should consider how such genes interact.

## Materials and methods

### Study subjects

The AMBER Consortium, described in detail elsewhere^45^ is a collaboration pooling data from four studies, the Carolina Breast Cancer Study (CBCS), the Women’s Circle of Health Study (WCHS), the Black Women’s Health Study (BWHS), and the Multiethnic Cohort (MEC). Briefly, the CBCS is a population-based case–control study of women aged 20 to 74 years that began in North Carolina in 1993.^46^ Cases were identified through the North Carolina Central Cancer Registry’s rapid case ascertainment system, and controls were enrolled through 2001 using Division of Motor Vehicles lists (age<65 years) and Health Care Financing Administration lists (age⩾65 years). Questionnaire data and samples for DNA analysis were obtained by interviewers in home visits. The WCHS is a case–control study that began in 2002 with ascertainment of cases aged 20 to 75 years from New York City hospitals, later expanding to ten counties in New Jersey, with case identification using the New Jersey State Cancer Registry’s rapid case ascertainment system.^47,48^ Controls have been recruited through random digit dialing as well as community-based efforts. In-person interviewers collect risk factor data and obtain samples for DNA analysis.

The BWHS is a prospective cohort study that began in 1995 when 59,000 African-American women 21–69 years of age from across the United States completed a postal health questionnaire.^49^ Breast cancer cases are identified by self-report in biennial follow-up questionnaires, and cases are confirmed by medical records or from state cancer registry data and the National Death Index. Approximately 27,000 BWHS participants have given saliva samples for DNA analysis. The MEC is a prospective cohort study in Hawaii and Southern California that began in 1993 with the enrollment of men and women aged 45–75 years.^50^ Data are collected through questionnaires mailed at 5-year intervals, and breast cancer cases are confirmed by linkage with the California and Hawaii state cancer registries and the National Death Index. Controls for BWHS and MEC were selected from among all non-cases in those studies.

The CBCS was approved by the Institutional Review Board at the University of North Carolina at Chapel Hill School of Medicine. The WCHS was approved by the Institutional Review Boards at the University of Medicine and Dentistry of New Jersey (presently Rutgers University), Mount Sinai School of Medicine, and Roswell Park Cancer Institute. The BWHS was approved by the Institutional Review Board at the Boston University School of Medicine. The MEC was approved by the Institutional Review Boards of the University of Hawaii and University of Southern California. Written informed consent was obtained from each participant.

Eligible cases for analysis were women with a first diagnosis of incident invasive breast cancer or ductal carcinoma *in situ*, with available DNA samples for genotyping. Determination of ER status for cases was based on pathology data obtained from state cancer registry records or directly from hospital records.

### Gene and SNP selection

We selected 184 genes in the insulin, IGF, GH, and LEP pathways from the Molecular Signature Database (MSigDB)^51^ (Supplementary Table 1). Tag SNPs were then selected for all 184 genes in order to capture (at *r*^2^⩾0.8) as many SNPs as possible with MAF⩾10%, based on the haplotype structure of the Yoruban population (YRI) in 1000 Genomes (http://www.1000genomes.org/).

### Genotyping and quality control

Genotyping using the Illumina Human Exome Beadchip v1.1 with custom content was performed by the Center for Inherited Disease Research (CIDR). The variants selected for this analysis were included as part of more than 159,000 custom content SNPs added to the Exome Beadchip to support the scientific goals of the AMBER consortium.

Of the 405,555 SNPs attempted for genotyping, 381,212 were released by CIDR and 299,873 of these remained after removing SNPs that were monomorphic, were positional duplicates, were on the Y chromosome, had Hardy–Weinberg Equilibrium *P*<1×10^−4^, had call rate<0.98, had >1 Mendelian errors in trios from HapMap (http://hapmap.ncbi.nlm.nih.gov), or had >2 discordant calls in duplicate samples. Genotypes were attempted for 6,936 study subjects from the BWHS, CBCS, and WCHS, and were completed with call rate >98% for 6,828 participants (3,130 cases and 3,698 controls). The University of Washington performed imputation using the IMPUTE2 software^52^ and the 1000 Genomes Phase I reference panel (5/21/2011 1000 Genomes data, December 2013).

Genetic data from 533 cases and 989 controls in the MEC study had been genotyped on the Illumina Human 1M-Duo array and SNPs were imputed from 1000 Genomes. Imputed genotypes from MEC were combined with imputed data from BWHS, CBCS, and WCHS into a final data set after additional quality control. Variants with mismatching alleles or allele frequencies that were different by >0.15 in MEC versus the other three studies were omitted. Also, SNPs with MAFs<0.5% or imputation score INFO<0.5 in either study were removed. After these exclusions, there were 91,627 genotyped or imputed SNPs with MAF⩾2% in the 184 genes of interest.

Genotype principal components were computed using the smartpca program in the EIGENSOFT package.^53^ Relationship checking using PLINK software^54^ (http://pngu.mgh.harvard.edu/~purcell/plink/) identified several relatives among and within the individual studies. Related individuals and those with more extreme principal components were flagged so that relationships could be taken into account and sensitivity analyses could be performed. The principal components of genotype were tested for association with case status after accounting for the study covariates: study, age (10-year groupings and matching variable), geographic region (matching variable), and DNA source (Oragene-saliva, blood and mouthwash-saliva). No principal components were strongly associated with case status after controlling for the study covariates. For case status and subtype association analyses, we included principal components that were associated with *P*<0.1 in the full covariate model.

### Statistical analysis

Gene-based association tests were conducted for the 184 selected genes. We used a multi-locus adaptive joint test^55^ as implemented in the R package AdaJoint. The test identifies the best subset of SNPs that jointly show the strongest evidence for association with disease in a given gene through a variable selection procedure that takes into account the LD structure. The significance level of the gene-based test is evaluated through a direct simulation approach that generates the null distribution of the statistic. Because the score test implemented in AdaJoint is not optimal for rare variants, we excluded SNPs with MAF<2%. To avoid missing independent association signals due to correlations between SNPs, we excluded the SNP with lower MAF from each SNP pair with correlation *r*^2^>0.9. These exclusions resulted in a final analytic list of 31,657 SNPs in 184 genes. Our analysis searched up to the best five most significant SNPs within each gene. In order to account for multiple testing, we set the alpha level for statistical significance at 3.0×10^−4^ (0.05/184 genes).

Odds ratios (ORs) and 95% confidence intervals (CIs) for the most significant SNPs in the identified genes were estimated using logistic regression (PLINK version 1.9).^54^ Models were adjusted for the covariates noted above and for genotype principal components 5, 6, and 8.

## Figures and Tables

**Table 1:** Characteristics of participants by study in the AMBER consortium

	*Study*				
	*BWHS*	*CBCS*	*WCHS*	*MEC*	*AMBER*^a^
Controls	2,249	615	834	989	4,687
Cases	901	1408	821	533	3,663
ER^+^ cases	498	741	435	309	1,983
ER^−^ cases	233	565	165	135	1,098
Unknown ER	170	102	221	89	582
					
*Age at diagnosis*					
<40	47	204	85	0	336
40–49	262	459	215	9	945
50–59	302	381	292	112	1,087
60–69	204	267	173	175	819
⩾70	86	97	56	237	476

Abbreviations: AMBER, African American Breast Cancer Epidemiology and Risk; BWHS, Black Women’s Health Study; CBCS, Carolina Breast Cancer Study; ER, estrogen receptor; MEC, Multi-Ethnic Cohort; WCHS, Women’s Circle of Health Study.

a AMBER includes all four studies: BWHS, CBCS, WCHS, and MEC.

**Table 2:** Association results of genes with *P*⩽0.01 with overall, ER^+^, and ER^−^ breast cancer risk in the AMBER Consortium

*Gene*	*Total number of SNPs*	*Effective number of SNPs*	P *value*		
			*All cases*	*ER*^+^	*ER*^−^
*BAIAP2*	652	258	3.0×10^−3^	1.0×10^−3^	0.039
*CALM2*	158	72	9.0×10^−3^	0.018	0.39
*CSNK2A1*	400	150	0.033	0.010	0.11
*BRAF*	812	106	0.013	0.40	3.0×10^−3^
*BAD*	51	26	0.049	0.32	4.9×10^−3^
*MAPK3*	24	11	0.39	0.88	9.0×10^−3^

**Table 3:** Single SNPs associations in genes with *P*⩽0.01

*Gene, SNP*	*Type*^a^	*INFO*^b^	*Alleles*^c^	*EAF*^d^ *(%)*	*OR (95% CI)*^e^, P *value*		
					*All cases (3,663)*	*ER*^+^* (1,983)*	*ER*^−^* (1,098)*
*BAIAP2*							
rs142882938	I	0.98	−/T	4.8	1.45 (1.25–1.69), 6.0×10^−7^	1.55 (1.31–1.84), 4.6×10^−7^	1.39 (1.11–1.72), 3.4×10^−3^
							
*CALM2*							
rs13032512	I	0.92	A/G	5.5	1.33 (1.15–1.54), 1.3×10^−4^	1.30 (1.09–1.55), 4.1×10^−3^	1.35 (1.08–1.68), 8.2×10^−3^
							
*CSNK2A1*							
rs434410	I	1.00	T/C	24.3	1.18 (1.09–1.27), 2.8×10^−5^	1.21 (1.11–1.33), 3.3×10^−5^	1.18 (1.05–1.33), 5.3×10^−3^
							
*BRAF*							
rs114729114	I	0.78	T/C	2.7	1.54 (1.24–1.92), 9.2×10^−5^	1.45 (1.11–1.88), 5.7×10^−3^	2.04 (1.50–2.77), 4.9×10^−6^
							
*BAD*							
rs2286615	I	1.00	A/G	4.3	0.75 (0.63–0.89), 1.3×10^−3^	0.78 (0.63–0.96), 0.018	0.60 (0.45–0.80), 5.0×10^−4^
chr11:64038448:I	I	0.68	CT/−	7.9	0.83 (0.71–0.97), 0.017	0.89 (0.74–1.07), 0.22	0.70 (0.55–0.89), 4.4×10^−3^
							
*MAPK3*							
rs78564187	G		A/G	18.0	1.07 (0.98–1.16), 0.13	1.03 (0.93–1.14), 0.58	1.26 (1.17–1.35), 3.7×10^−4^

a SNP type; imputed (I) or genotyped (G).

b INFO score for imputed SNPs.

c Effect allele/reference allele.

d Effect allele frequency in AMBER.

e Adjusted for study site, age (10-year groupings), geographic region, DNA source (saliva, blood, and mouthwash), and genotype principal components 5, 6, 8.
