# Supplementary material for: Genetic variation in the insulin, insulin-like growth factor, growth hormone, and leptin pathways in relation to breast cancer in African-American women: the AMBER consortium
Source: NPJ Breast Cancer. 2016 Oct 26;2:16034–. doi: 10.1038/npjbcancer.2016.34 (PMC5142758; doi:10.1038/npjbcancer.2016.34)
Supplement: Supplementary Table 1 [file npjbcancer201634-s1.doc]

| Supplementary Table 1. List of selected genes and P-value of gene-based tests | | | | | |
| --- | --- | --- | --- | --- | --- |
| Gene | Total number of SNPs | Effective number of SNPs | P-value of gene-based test | | |
|  |  |  | All cases | ER+ | ER- |
| *CALML6* | 74 | 42 | 0.724 | 0.711 | 0.819 |
| *PRKCZ* | 1056 | 382 | 0.116 | 0.657 | 0.253 |
| *PIK3CD* | 362 | 166 | 0.577 | 0.159 | 0.747 |
| *MTOR* | 845 | 132 | 0.082 | 0.171 | 0.098 |
| *PTPRF* | 660 | 211 | 0.934 | 0.956 | 0.902 |
| *PIK3R3* | 492 | 99 | 0.107 | 0.329 | 0.109 |
| *MKNK1* | 256 | 88 | 0.213 | 0.332 | 0.924 |
| *PRKAA2* | 456 | 95 | 0.347 | 0.351 | 0.838 |
| *JUN* | 87 | 43 | 0.138 | 0.604 | 0.259 |
| *PRKACB* | 819 | 136 | 0.272 | 0.9 | 0.256 |
| *NRAS* | 72 | 23 | 0.714 | 0.065 | 0.842 |
| *PRKAB2* | 172 | 47 | 0.92 | 0.647 | 0.868 |
| *SHC1* | 31 | 15 | 0.121 | 0.127 | 0.424 |
| *PKLR* | 71 | 30 | 0.832 | 0.425 | 0.791 |
| *PAPPA2* | 1558 | 444 | 0.876 | 0.151 | 0.876 |
| *CACNA1E* | 1771 | 621 | 0.71 | 0.069 | 0.738 |
| *AKT3* | 1334 | 324 | 0.631 | 0.332 | 0.928 |
| *PPP1CB* | 316 | 64 | 0.846 | 0.702 | 0.208 |
| *SOS1* | 691 | 170 | 0.638 | 0.395 | 0.557 |
| *PRKCE* | 3842 | 1742 | 0.884 | 0.179 | 0.5 |
| *RHOQ* | 117 | 52 | 0.634 | 0.697 | 0.166 |
| *CALM2* | 158 | 72 | 0.009 | 0.018 | 0.387 |
| *HK2* | 480 | 220 | 0.013 | 0.036 | 0.315 |
| *G6PC2* | 188 | 104 | 0.298 | 0.324 | 0.975 |
| *IGFBP2* | 263 | 125 | 0.756 | 0.398 | 0.717 |
| *IGFBP5* | 166 | 74 | 0.464 | 0.21 | 0.552 |
| *PRKAG3* | 104 | 42 | 0.856 | 0.884 | 0.642 |
| *IRS1* | 373 | 126 | 0.976 | 0.623 | 0.712 |
| *EIF4E2* | 296 | 77 | 0.674 | 0.927 | 0.303 |
| *INPP5D* | 1101 | 469 | 0.508 | 0.646 | 0.084 |
| *RAF1* | 558 | 93 | 0.072 | 0.113 | 0.837 |
| *PRKAR2A* | 214 | 42 | 0.922 | 0.814 | 0.876 |
| *PRKCD* | 298 | 129 | 0.136 | 0.164 | 0.604 |
| *CACNA1D* | 1853 | 823 | 0.379 | 0.95 | 0.119 |
| *CBLB* | 1213 | 191 | 0.856 | 0.988 | 0.177 |
| *GSK3B* | 1284 | 105 | 0.394 | 0.336 | 0.832 |
| *PIK3CB* | 439 | 79 | 0.041 | 0.536 | 0.303 |
| *PRKCI* | 655 | 165 | 0.474 | 0.62 | 0.208 |
| *SLC2A2* | 224 | 58 | 0.482 | 0.169 | 0.965 |
| *PIK3CA* | 357 | 63 | 0.06 | 0.374 | 0.243 |
| *ADIPOQ* | 207 | 97 | 0.296 | 0.253 | 0.316 |
| *PPARGC1A* | 696 | 271 | 0.671 | 0.838 | 0.179 |
| *MAPK10* | 1898 | 408 | 0.802 | 0.845 | 0.323 |
| *EIF4E* | 375 | 53 | 0.916 | 0.989 | 0.65 |
| *GAB1* | 745 | 204 | 0.375 | 0.327 | 0.916 |
| *PRKAA1* | 276 | 52 | 0.638 | 0.75 | 0.918 |
| *PIK3R1* | 538 | 213 | 0.099 | 0.385 | 0.39 |
| *RASA1* | 451 | 63 | 0.881 | 0.649 | 0.453 |
| *AP3S1* | 423 | 120 | 0.753 | 0.704 | 0.612 |
| *EIF4E1B* | 98 | 51 | 0.158 | 0.012 | 0.598 |
| *HK3* | 108 | 46 | 0.584 | 0.583 | 0.339 |
| *MAPK9* | 559 | 217 | 0.773 | 0.907 | 0.759 |
| *FLOT1* | 134 | 28 | 0.441 | 0.191 | 0.107 |
| *TNF* | 24 | 14 | 0.592 | 0.957 | 0.433 |
| *SRF* | 78 | 32 | 0.302 | 0.565 | 0.771 |
| *PLG* | 482 | 175 | 0.798 | 0.382 | 0.328 |
| *PRKAR1B* | 1392 | 504 | 0.695 | 0.216 | 0.768 |
| *GCK* | 300 | 122 | 0.491 | 0.431 | 0.916 |
| *IGFBP1* | 158 | 55 | 0.968 | 0.798 | 0.246 |
| *IGFBP3* | 116 | 50 | 0.788 | 0.81 | 0.566 |
| *GRB10* | 1493 | 472 | 0.68 | 0.391 | 0.174 |
| *PHKG1* | 65 | 29 | 0.924 | 0.515 | 0.844 |
| *SH2B2* | 264 | 124 | 0.958 | 0.982 | 0.266 |
| *PIK3CG* | 243 | 91 | 0.335 | 0.714 | 0.154 |
| *PRKAR2B* | 600 | 180 | 0.582 | 0.755 | 0.166 |
| *PPP1R3A* | 284 | 60 | 0.37 | 0.91 | 0.101 |
| *BRAF* | 812 | 106 | 0.013 | 0.405 | 0.003 |
| *RHEB* | 413 | 137 | 0.959 | 0.967 | 0.303 |
| *PRKAG2* | 2796 | 1267 | 0.879 | 0.976 | 0.034 |
| *PPP1R3B* | 217 | 101 | 0.97 | 0.938 | 0.146 |
| *EIF4EBP1* | 234 | 56 | 0.138 | 0.569 | 0.417 |
| *IKBKB* | 289 | 80 | 0.237 | 0.149 | 0.791 |
| *MAFA* | 176 | 99 | 0.512 | 0.374 | 0.977 |
| *RPS6* | 147 | 33 | 0.807 | 0.934 | 0.718 |
| *PRKACG* | 69 | 36 | 0.652 | 0.435 | 0.753 |
| *CTSL1* | 161 | 57 | 0.554 | 0.94 | 0.129 |
| *SHC3* | 1006 | 267 | 0.59 | 0.596 | 0.27 |
| *FBP2* | 235 | 94 | 0.482 | 0.675 | 0.833 |
| *FBP1* | 460 | 154 | 0.754 | 0.83 | 0.243 |
| *PAPPA* | 1161 | 440 | 0.46 | 0.425 | 0.876 |
| *RAPGEF1* | 893 | 252 | 0.025 | 0.214 | 0.098 |
| *TSC1* | 217 | 66 | 0.172 | 0.436 | 0.261 |
| *CACNA1B* | 1069 | 409 | 0.331 | 0.036 | 0.742 |
| *CALML5* | 149 | 72 | 0.549 | 0.587 | 0.153 |
| *CALML3* | 81 | 49 | 0.233 | 0.221 | 0.355 |
| *MAPK8* | 763 | 97 | 0.122 | 0.061 | 0.181 |
| *HK1* | 1141 | 441 | 0.227 | 0.174 | 0.206 |
| *PPP1R3C* | 162 | 44 | 0.535 | 0.668 | 0.181 |
| *SORBS1* | 1620 | 582 | 0.144 | 0.261 | 0.697 |
| *HRAS* | 120 | 49 | 0.639 | 0.652 | 0.666 |
| *IGF2* | 186 | 99 | 0.497 | 0.491 | 0.478 |
| *INS* | 19 | 14 | 0.247 | 0.868 | 0.023 |
| *PDE3B* | 614 | 102 | 0.134 | 0.02 | 0.277 |
| *KCNJ11* | 73 | 34 | 0.715 | 0.945 | 0.334 |
| *ABCC8* | 717 | 329 | 0.628 | 0.893 | 0.819 |
| *F2* | 155 | 46 | 0.344 | 0.806 | 0.013 |
| *BAD* | 51 | 26 | 0.049 | 0.325 | 0.005 |
| *PYGM* | 62 | 36 | 0.098 | 0.617 | 0.016 |
| *PPP1CA* | 78 | 28 | 0.056 | 0.322 | 0.02 |
| *RPS6KB2* | 27 | 10 | 0.55 | 0.733 | 0.233 |
| *MMP1* | 11 | 7 | 0.992 | 0.989 | 0.648 |
| *CBL* | 435 | 101 | 0.735 | 0.839 | 0.595 |
| *CACNA1C* | 3894 | 1650 | 0.671 | 0.785 | 0.283 |
| *PDE3A* | 2180 | 876 | 0.83 | 0.632 | 0.664 |
| *GYS2* | 490 | 152 | 0.987 | 0.984 | 0.801 |
| *KRAS* | 334 | 98 | 0.839 | 0.819 | 0.6 |
| *PRKAG1* | 49 | 23 | 0.185 | 0.493 | 0.608 |
| *IGFBP6* | 76 | 32 | 0.536 | 0.109 | 0.81 |
| *SOCS2* | 74 | 31 | 0.19 | 0.139 | 0.207 |
| *IGF1* | 323 | 110 | 0.597 | 0.692 | 0.336 |
| *ACACB* | 956 | 395 | 0.372 | 0.24 | 0.617 |
| *PPP1CC* | 176 | 48 | 0.561 | 0.677 | 0.083 |
| *PTPN11* | 296 | 67 | 0.48 | 0.252 | 0.662 |
| *PRKAB1* | 120 | 34 | 0.515 | 0.699 | 0.851 |
| *PDX1* | 170 | 45 | 0.926 | 0.895 | 0.549 |
| *FOXO1* | 876 | 296 | 0.748 | 0.98 | 0.866 |
| *IRS2* | 311 | 155 | 0.682 | 0.635 | 0.381 |
| *PCK2* | 31 | 16 | 0.59 | 0.218 | 0.487 |
| *SOS2* | 687 | 135 | 0.367 | 0.325 | 0.92 |
| *PYGL* | 322 | 119 | 0.628 | 0.93 | 0.554 |
| *SOCS4* | 106 | 32 | 0.274 | 0.43 | 0.257 |
| *FOS* | 106 | 61 | 0.823 | 0.868 | 0.305 |
| *CALM1* | 168 | 84 | 0.598 | 0.903 | 0.942 |
| *AKT1* | 257 | 120 | 0.119 | 0.227 | 0.8 |
| *SHC4* | 851 | 331 | 0.267 | 0.334 | 0.306 |
| *MAP2K1* | 685 | 111 | 0.448 | 0.485 | 0.263 |
| *PKM2* | 143 | 47 | 0.768 | 0.848 | 0.962 |
| *IGF1R* | 2171 | 900 | 0.255 | 0.659 | 0.509 |
| *IGFALS* | 198 | 62 | 0.142 | 0.043 | 0.848 |
| *TSC2* | 256 | 115 | 0.051 | 0.026 | 0.76 |
| *PDPK1* | 146 | 47 | 0.39 | 0.204 | 0.207 |
| *SOCS1* | 166 | 76 | 0.178 | 0.541 | 0.18 |
| *MAPK3* | 24 | 11 | 0.392 | 0.878 | 0.009 |
| *PHKG2* | 49 | 17 | 0.745 | 0.761 | 0.997 |
| *PHKB* | 616 | 69 | 0.596 | 0.487 | 0.847 |
| *MMP2* | 314 | 135 | 0.142 | 0.579 | 0.175 |
| *BCAR1* | 413 | 159 | 0.596 | 0.516 | 0.577 |
| *FOXC2* | 188 | 95 | 0.522 | 0.947 | 0.194 |
| *CRK* | 375 | 113 | 0.077 | 0.019 | 0.346 |
| *INPP5K* | 179 | 77 | 0.598 | 0.152 | 0.93 |
| *SLC2A4* | 88 | 35 | 0.114 | 0.224 | 0.14 |
| *PIK3R5* | 759 | 310 | 0.588 | 0.222 | 0.76 |
| *SREBF1* | 152 | 82 | 0.984 | 0.991 | 0.866 |
| *FLOT2* | 92 | 38 | 0.388 | 0.599 | 0.852 |
| *ACACA* | 1226 | 318 | 0.652 | 0.167 | 0.865 |
| *IGFBP4* | 154 | 64 | 0.128 | 0.292 | 0.927 |
| *G6PC* | 127 | 62 | 0.509 | 0.522 | 0.061 |
| *CACNA1G* | 344 | 165 | 0.193 | 0.093 | 0.031 |
| *RPS6KB1* | 208 | 50 | 0.131 | 0.329 | 0.689 |
| *PRKAR1A* | 163 | 41 | 0.03 | 0.134 | 0.052 |
| *GRB2* | 607 | 110 | 0.441 | 0.054 | 0.185 |
| *EXOC7* | 220 | 62 | 0.28 | 0.075 | 0.893 |
| *SOCS3* | 127 | 78 | 0.748 | 0.537 | 0.861 |
| *RPTOR* | 3423 | 1109 | 0.252 | 0.062 | 0.157 |
| *BAIAP2* | 652 | 258 | 0.003 | 0.001 | 0.039 |
| *FASN* | 180 | 106 | 0.919 | 0.262 | 0.938 |
| *SHC2* | 582 | 311 | 0.549 | 0.86 | 0.706 |
| *MKNK2* | 122 | 74 | 0.857 | 0.994 | 0.553 |
| *MAP2K2* | 324 | 144 | 0.901 | 0.98 | 0.042 |
| *TRIP10* | 64 | 41 | 0.275 | 0.635 | 0.555 |
| *INSR* | 1595 | 763 | 0.353 | 0.314 | 0.708 |
| *CACNA1A* | 2161 | 999 | 0.713 | 0.548 | 0.894 |
| *PRKACA* | 99 | 38 | 0.741 | 0.55 | 0.133 |
| *PIK3R2* | 92 | 39 | 0.565 | 0.719 | 0.849 |
| *AKT2* | 241 | 58 | 0.527 | 0.514 | 0.78 |
| *LIPE* | 17 | 7 | 0.088 | 0.199 | 0.716 |
| *CBLC* | 282 | 148 | 0.2 | 0.338 | 0.895 |
| *CALM3* | 96 | 50 | 0.502 | 0.959 | 0.088 |
| *GYS1* | 175 | 77 | 0.897 | 0.974 | 0.339 |
| *KLK3* | 270 | 135 | 0.474 | 0.936 | 0.316 |
| *CSNK2A1* | 400 | 150 | 0.033 | 0.01 | 0.115 |
| *PYGB* | 476 | 108 | 0.166 | 0.155 | 0.089 |
| *PTPN1* | 381 | 86 | 0.3 | 0.364 | 0.421 |
| *PCK1* | 186 | 96 | 0.755 | 0.88 | 0.852 |
| *PPP1R3D* | 3 | 2 | 0.875 | 0.863 | 0.563 |
| *CRKL* | 184 | 41 | 0.355 | 0.356 | 0.807 |
| *MAPK1* | 692 | 124 | 0.934 | 0.43 | 0.65 |
| *ARAF* | 75 | 46 | 0.503 | 0.488 | 0.207 |
| *ELK1* | 53 | 20 | 0.825 | 0.575 | 0.675 |
| *FOXO4* | 63 | 40 | 0.964 | 0.758 | 0.401 |
| *IRS4* | 60 | 23 | 0.753 | 0.938 | 0.592 |
| *PHKA1* | 381 | 84 | 0.461 | 0.886 | 0.118 |
| *PHKA2* | 249 | 62 | 0.804 | 0.944 | 0.396 |
| *PRKX* | 874 | 521 | 0.328 | 0.195 | 0.06 |
